# Tumor suppressor miR-218 directly targets epidermal growth factor receptor (EGFR) expression in triple-negative breast cancer, sensitizing cells to irradiation

**DOI:** 10.1007/s00432-023-04750-x

**Published:** 2023-04-23

**Authors:** Franz-Josef Wischmann, Fabian M. Troschel, Maj Frankenberg, Björn Kemper, Archana Vijaya Kumar, Mark Sicking, Sherif Abdelaziz Ibrahim, Ludwig Kiesel, Martin Götte, Hans Theodor Eich, Burkhard Greve

**Affiliations:** 1 Department of Radiation Oncology, University Hospital Münster, Albert-Schweitzer-Campus 1, Gebäude A1, 48149 Münster, Germany; 2 Biomedical Technology Center, Medical Faculty, University of Münster, Münster, Germany; 3 Department of Gynecology and Obstetrics, University Hospital Münster, Münster, Germany; 4 Department of Zoology, Faculty of Science, Cairo University, Giza, 12613 Egypt

**Keywords:** miR-218, EGFR, Radiotherapy, Triple-negative breast cancer, Mitosis, Invasiveness

## Abstract

**Purpose:**

MicroRNA-218 (miR-218) is a key regulator of numerous processes relevant to tumor progression. In the present study, we aimed to characterize the relationship between miR-218 and the Epidermal Growth Factor Receptor (EGFR) as well as to understand downstream effects in triple-negative breast cancer (TNBC).

**Methods:**

We assessed miR-218 and EGFR expression in cell lines and publicly available primary breast cancer gene expression data. We then overexpressed miR-218 in two TNBC cell lines and investigated effects on EGFR and downstream mitogen-activated protein (MAP) kinase signaling. Luciferase reporter assay was used to characterize a direct binding interaction between miR-218 and *EGFR* mRNA. Digital holographic microscopy helped investigate cell migration and dry mass after miR-218 overexpression. Cell division and invasion were assessed microscopically, while radiation response after miR-218 overexpression alone or combined with additional EGFR knockdown was investigated via clonogenic assays.

**Results:**

We found an inverse correlation between *EGFR* expression and miR-218 levels in cell lines and primary breast cancer tissues. MiR-218 overexpression resulted in a downregulation of EGFR via direct binding of the mRNA. Activation of EGFR and downstream p44/42 MAPK signaling were reduced after pre-miR-218 transfection. Cell proliferation, motility and invasiveness were inhibited whereas cell death and mitotic catastrophe were upregulated in miR-218 overexpressing cells compared to controls. MiR-218 overexpressing and *EGFR* siRNA-treated cells were sensitized to irradiation, more than miR-218 overexpressing cells alone.

**Conclusion:**

This study characterizes the antagonistic relationship between miR-218 and EGFR. It also demonstrates downstream functional effects of miR-218 overexpression, leading to anti-tumorigenic cellular changes.

**Supplementary Information:**

The online version contains supplementary material available at 10.1007/s00432-023-04750-x.

## Background

MicroRNAs (miRNAs) belong to the non-coding class of short interfering RNAs. They are pivotal post-transcriptional gene expression regulators as they bind specific mRNA sequences, thus blocking translation. Hence, they orchestrate a multitude of physiological and pathological processes (Ibrahim et al. 2014). Within this diverse class, some miRNAs have been identified to possess key regulatory functions in malignancies, including for chemo- (Frixa et al. 2015) and radioresistance (Troschel et al. 2018).

MiR-218 was found to play significant roles in tumorigenesis and cancer progression both in vitro and in vivo (Lu et al. 2015). In different tumor entities, including breast cancer, a high expression of miR-218 in primary patient tissues is associated with a good prognosis (Li et al. 2015a, b; Liu et al. 2015; Ahmadinejad et al. 2017). Subsequently, additional studies have been performed to further elucidate the tumor-suppressing capacity of miR-218 in breast cancer in vitro (Liu et al. 2016), yet research is ongoing.

The Epidermal Growth Factor Receptor (EGFR) has previously been identified to be directly regulated by miR-218 in non-small cell lung cancer in vitro and in vivo (Zhu et al. 2016) and glioblastoma in vitro (Mathew et al. 2015), and a subsequent study has been able to establish links to therapy resistance (Jin et al. 2020). However, a recent investigation suggested a co-expression of EGFR and miR-218 in breast cancer (Qian et al. 2021).

Given the high therapeutic relevance of EGFR-related pathways and the lack of understanding regarding the interplay of miR-218 and EGFR in breast cancer, we aimed to determine the relation between miR-218, EGFR and therapy resistance in breast cancer. Additionally, we set out to investigate additional miR-218 targets and to provide evidence for miR-218-mediated changes using digital holographic microscopy.

## Methods

### Cell culture

The human cell lines used in this study were obtained from LGC-Promochem/ATCC (Wesel, Germany). The hormone receptor positive cell line MCF-7 was cultivated in RPMI (Merck-Millipore, Darmstadt, Germany) medium supplemented with 10% FCS (Biochrome™, Merck, Darmstadt, Germany) and 1% Pen/Strep (Gibco™, ThermoFisher Scientific, Waltham, MA, USA), while the triple-negative cell lines SK-BR-3, MDA-MB-231, HCC1806, BT549 and MDA-MB-468 as well as the lung cancer cell line A549 and the keratinocyte cell line HaCaT were cultured in DMEM medium (Sigma, Merck-Millipore, Darmstadt, Germany) supplemented with 10% FCS and 1% Pen/Strep. The breast cancer cell line BT474 was cultured in RPMI under addition of 20% FCS and 1% Pen/Strep as well as 0.1% insulin (Sigma, Merck-Millipore). MCF-10A, an immortalized breast epithelial cell line, was cultured in a 1:1 mixture of DMEM/F-12 (Merck-Millipore) supplemented with 5% horse serum, 1% Pen/Strep, 2 ng/mL EGF (Sigma, Merck-Millipore), 0.5 mg/mL Hydrocortisone (Sigma), 100 ng/mL Cholera toxin (Sigma) and 10 µg/mL Insulin. The esophageal cancer cell line K180 was cultured in RPMI 1640 medium without phenol red containing 10% FCS, 1% Pen/Strep and 25 mM HEPES. All cell lines were cultivated in a humidified atmosphere with 5% CO_2_ at 37 °C and regularly tested negative for mycoplasmas (Mycoplasma PCR test kit, Cytecs, Münster, Germany).

### Cell transfection

For transient transfection experiments, 100,000 cells were seeded in each well of a 6-well plate (Greiner, Solingen, Germany) 24 h before treatment. Transfection was performed according to the manufacturer’s instructions by using 2µL Lipofectamine^®^ RNAiMAX (Invitrogen™, ThermoFisher Scientific) mixed in OptiMEM media (Gibco™) with addition of 2µL *EGFR* siRNA (10 pM) or pre-miR-218 (10 pM) or pre-miR negative control (10 pM) per well, respectively. Twenty-four hours after transfection, OptiMEM was replaced by regular culture medium. SiRNAs and pre-miR molecules can be found in Supplementary Table 1.

### Total RNA and microRNA isolation and reverse transcription

Cells were harvested with 0.05% Trypsin, centrifuged and washed with PBS before lysis. For total RNA isolation the Qiagen RNeasy^®^ Mini Kit (Qiagen, Hilden, Germany) was used. MiRNA was isolated with the mirVana™ Kit from Ambion™ (ThermoFisher Scientific) according to the manufacturer’s instruction. Isolated RNAs were stored at − 70 °C.

For reverse transcriptase reaction, 1 µg of total RNA was reverse transcribed into cDNA using random primers with the ABI High Capacity cDNA Reverse Transcription Kit (ABI, ThermoFisher Scientific). The TaqMan^®^ MicroRNA Reverse Transcription Kit (ThermoFisher Scientific) was used for reverse transcription of 100 ng microRNA with miR-specific primer systems according to the kit’s guidelines.

### Quantitative real-time PCR (qPCR)

Analyses were run as previously described for mRNA and miRNA (Eggers et al. 2016). For mRNA, the SYTO9 system (ThermoFisher Scientific) was used according to manufacturer’s instructions. *HPRT* (hypoxanthine–guanine-phosphoribosyltransferase) was used as reference for normalization. For miRNA, TaqMan^®^ assays were used, normalizing to *RNU6B* as a reference. Analyses were run on a Rotor-Gene Q machine (Qiagen). Data were expressed as fold change using the 2^–ΔΔCT^ method. Primers are listed in Supplementary Table 2. *EGFR* primers were synthesized by Eurogentech (Köln, Germany).

### Luciferase assay

Posttranscriptional regulation of *EGFR* expression by miR-218 was determined in MDA-MB-231 cells by using the EGFR-specific Luc-Pair miR Luciferase Assay from GeneCopoeia™ (Rockville, MD, USA) (Product ID: HmiT004605-MT01), expressing firefly luciferase under the control of the human EGFR 3′UTR, and renilla luciferase under the control of the cytomegaly virus promoter as an internal control for transfection efficiency. 6-well plates were seeded with 300,000 cells per well. Cells were then transfected using 2µL lipofectamine, 0.5µL plasmid (100 ng) and 4 µl pre-miR-218 (20 pmol) or control-pre-miRNA in serum and antibiotic-free OptiMEM medium. After 24 h the transfection medium was replaced by culture medium. 72 h after transfection the cells were lysed in the plate, the suspension was transferred into a 96-well plates, and mixed with the luciferase substrates. Luciferase activity was determined using a Veritas Microplate Luminometer (Turner BioSystems, Sunnyvale, USA), normalizing firefly luciferase activity to renilla activity.

### Western blotting

Western blot analysis was performed as described earlier (Greve et al. 2012). After protein transfer, the membrane was blocked for 1 h with 2.5% (w/v) dry milk (Cell Signaling, Cambridge, UK) in Tris-buffered saline with Tween20 (TBST). Primary antibodies (rabbit anti EGFR, rabbit anti p44/42 MAPK, rabbit anti phospho-p44/42 MAPK (Cell Signaling) all 1:2000, mouse anti β-actin (Sigma) 1:5000) were incubated overnight at 4 °C. After washing with TBST, the secondary antibodies goat anti-mouse-HRP at dilution 1:1000 and goat anti-rabbit-HRP at dilution 1:1000 (R&D, Minneapolis, USA) were incubated for 1 h at RT. Signals were detected using ECL substrate (Thermo Fisher Scientific, Langenselbold, Germany), and the bands were analysed using a Fusion SL System (VWR, Darmstadt, Germany).

### Flow cytometry

For flow cytometric analysis, 10^6^ cells were harvested by detachment with 2 mM EDTA. Cells were washed with PBS, incubated with 100 µL 0.1% (w/v) BSA (Roth) in PBS and, subsequently, with antibody (rat anti-EGFR (Abcam) 1:100). EGFR antibody binding was visualized with goat anti rat-Alexa488 (Dianova, Hamburg, Germany) 1:100. The flow cytometric analysis was performed on a CyFlow^®^ space flow cytometer (Partec, Münster, Germany). FloMax software was used for data evaluation (Quantum Analysis, Münster, Germany).

### Mitotic catastrophes

A total of 10^5^ cells were cultured on a cover slip in a 6-well plate. Cells were transfected as described above and fixed 24 h after transfection using 4% (w/v) PFA for 10 min. DNA was stained with DAPI (4′,6-diamidino-2-phenylindole) and the cytoskeleton with phalloidin-Alexa488 (Invitrogen™, Thermo Fisher Scientific). About 300 nuclei were documented and manually quantified for mitotic catastrophes per experiment. Fluorescence emission was determined by using a LSM880 microscope (Zeiss).

For live observation of mitotic catastrophes, the transfected and control MDA-MB-231 cells were seeded into 35 mm petri dishes (µ-Dish, ibidi, Munich, Germany) and medium was exchanged to DMEM + 10% FCS + 20 mM HEPES (BiochromTM, Berlin, Germany). Cell division was visualized by staining of tubulin (CellLight Tubulin-GFP, BacMam2.0, InvitrogenTM, Thermo Fisher Scientific) and chromosomes (Hoechst 33342) utilizing a Zeiss Axio Observer A1 microscope equipped with a 63 × microscope objective (Zeiss LD Plan-Neofluar 63x /0.75 Korr), a Retiga 2000 camera (QImaging, Surrey, Canada) and a heating chamber (HT200, Ibidi).

### Digital holographic microscopy (DHM)

Time-lapse observation with DHM was performed as previously described (Eggers et al. 2016). 2.5 × 10^4^ transiently transfected MDA-MB-231 cells were seeded into petri dishes (µ-Dish with glass lid, Ibidi). 24 h after transfection medium was exchanged to DMEM + 10% FCS + 20 mM HEPES (BiochromTM). Digital holograms were recorded every 3 min for 34 h. Cells were tracked in the retrieved series of quantitative DHM phase images by custom built software for automated cell tracking based on a previously described algorithm (Kemper et al. 2010). To quantify cell motility, the average migration distance was determined from the retrieved cell migration trajectories. Moreover, from the migration pattern the mean squared displacement (MSD) was calculated as previously described (Sridharan et al. 2011). In order to analyse cell growth and proliferation, cellular dry mass was retrieved by image segmentation using the free software cell profiler (www.cellprofiler.org) as described in Bettenworth et al. (2014).

### Cell invasion assay

Invasion was quantified as previously described (Troschel et al. 2020). Briefly, 25,000 cells were seeded on a Matrigel-coated 8.0 µm PET membrane as part of a Corning^®^ BioCoat™ Matrigel^®^ Invasion Chamber (Corning, New York, NY, USA) with 10% FCS medium. After 24 h of incubation, FCS was entirely removed from the cultivation chamber while 10% FCS was supplemented to the lower cell-free invasion chamber to generate a chemotactic gradient. Cells that invaded into the invasion chamber were fixed, stained and counted 24 h later.

### Clonogenic survival post-irradiation

To determine radiosensitizing effect of treatment, clonogenic assays were performed as detailed before (Falke et al. 2022). Cells were irradiated with 2 Gy 24 h after transfection with a TrueBeam linear accelerator (Varian Medical Systems, Palo Alto, CA, USA). Cells were then harvested, and pre-defined numbers of cells were seeded in 6-well plates and medium was supplemented. After 12 days of incubation, colonies (groups of more than 20 cells) were counted microscopically (Olympus CKX41 microscope; Olympus, Shinjuku, Tokyo, Japan). Plating efficiencies were calculated first. Then, survival fractions were calculated as plating efficiency after irradiation/plating efficiency without irradiation.

### Database analyses

We used two RNA sequencing datasets from primary breast cancer specimens from the GEO database: In the GSE58215 dataset, gene expression analysis was performed in 283 breast cancer specimens for mRNA and microRNA, allowing expression correlations (Aure et al. 2015). Here, normalized data as provided by the authors was directly correlated. In the GSE65505 dataset (Horton et al. 2015) we used 26 paired breast cancer samples pre- and post-irradiation. We pooled all different radiation regimens into the “post-radiation” group, while non-irradiated samples constituted the “pre-irradiation” group. Again, normalized gene expression data as provided by the authors was used for analysis.

### Statistical analysis

We used Spearman correlations to test for correlations, presenting spearman’s rho and the respective *p* value. We used paired t tests for pre- and post-irradiation comparisons in gene expression. For in vitro data, Student’s unpaired t-test was used. Here, all experiments consisted of at least three independent replicates. Statistical significance was set at *p* < 0.05 and error bars represent standard error of the mean. Analyses were performed and graphs were generated using Prism 8 (GraphPad Software, San Diego, CA, USA).

## Results

### The expression levels of EGFR and miR-218 are inversely correlated in different cancer cell lines and primary patient samples

The regulatory function of microRNAs is mediated by binding of their seed site to the 3′UTR of a target mRNA (Didiano and Hobert 2008). The Target Scan Human 6.2 algorithm predicts a 7mer seed sequence on miR-218 for *EGFR*, indicating a direct binding between miR-218 and *EGFR* (Supplementary Table 3). The probability of conserved targeting (pCT) estimates the probability of a specific binding event (Friedman et al. 2009). In the case of *EGFR*, the pCT is low, suggesting only moderate binding ability.

**Fig. 1:**
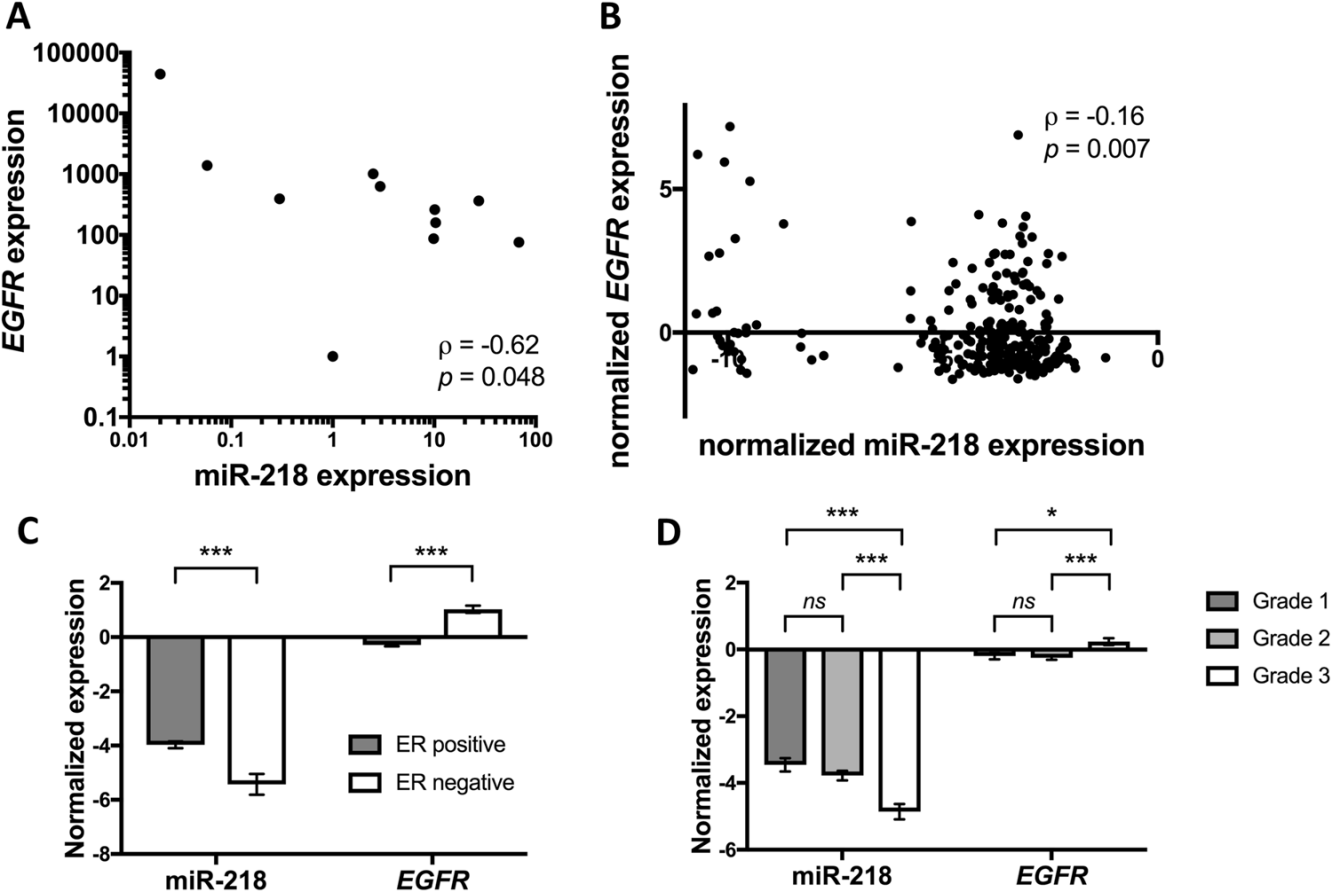
Expression of *EGFR* and miR-218 in cancer cell lines and primary breast cancer tissues. **A** *EGFR* and miR-218 show a negative expression correlation in eleven cancer cell lines. **B** *EGFR* and miR-218 show a negative expression correlation in primary breast cancer samples from the GSE58215 dataset. **C** miR-218 is expressed at higher levels in estrogen receptor (ER)-positive tumors, while *EGFR* is overexpressed in ER-negative tumors from the dataset (*p* < 0.001). **D** miR-218 is expressed at higher levels in grade 1 tumors while *EGFR* is overexpressed in grade 3 tumors from the dataset. *p < 0.05; **p < 0.01; ***p < 0.001

To understand the interplay between *EGFR* and miR-218, we then screened 11 cell lines for *EGFR* expression. The EGFR-negative cell line MCF-7 was chosen as a reference and compared to the remaining cell lines using the fold change 2^−ΔΔct^ method (Livak and Schmittgen 2001). The highest *EGFR* expression was determined for MDA-MB-468 followed by K180, MCF-10, HCC1806, SK-BR-3, MDA-MB-231, HaCat, A549, BT474, and BT549, respectively. There was a significant negative correlation between *EGFR* and miR-218 expression (*p* = 0.048, Spearman’s ρ =  − 0.62, Fig. 1A). We subsequently examined the relationship between miR-218 and *EGFR* using the sequencing analyses provided in the GSE58215 dataset. Here, we also found a negative association in 283 breast cancer samples (*p* = 0.007, Spearman’s ρ =  − 0.16, Fig. 1B). In subsequent analyses in the dataset, we found that miR-218 was overexpressed in estrogen receptor (ER)-positive tumors while *EGFR* was overexpressed in ER-negative samples (Fig. 1C). Similarly, miR-218 was expressed highest in low-grade tumors (histologic grade 1 or 2), while *EGFR* was primarily expressed in high-grade breast cancer samples (histologic grade 3, Fig. 1D).

### MiR-218 directly targets EGFR in cancer cells and perturbs activation of the downstream p44/42 MAPK

**Fig. 2:**
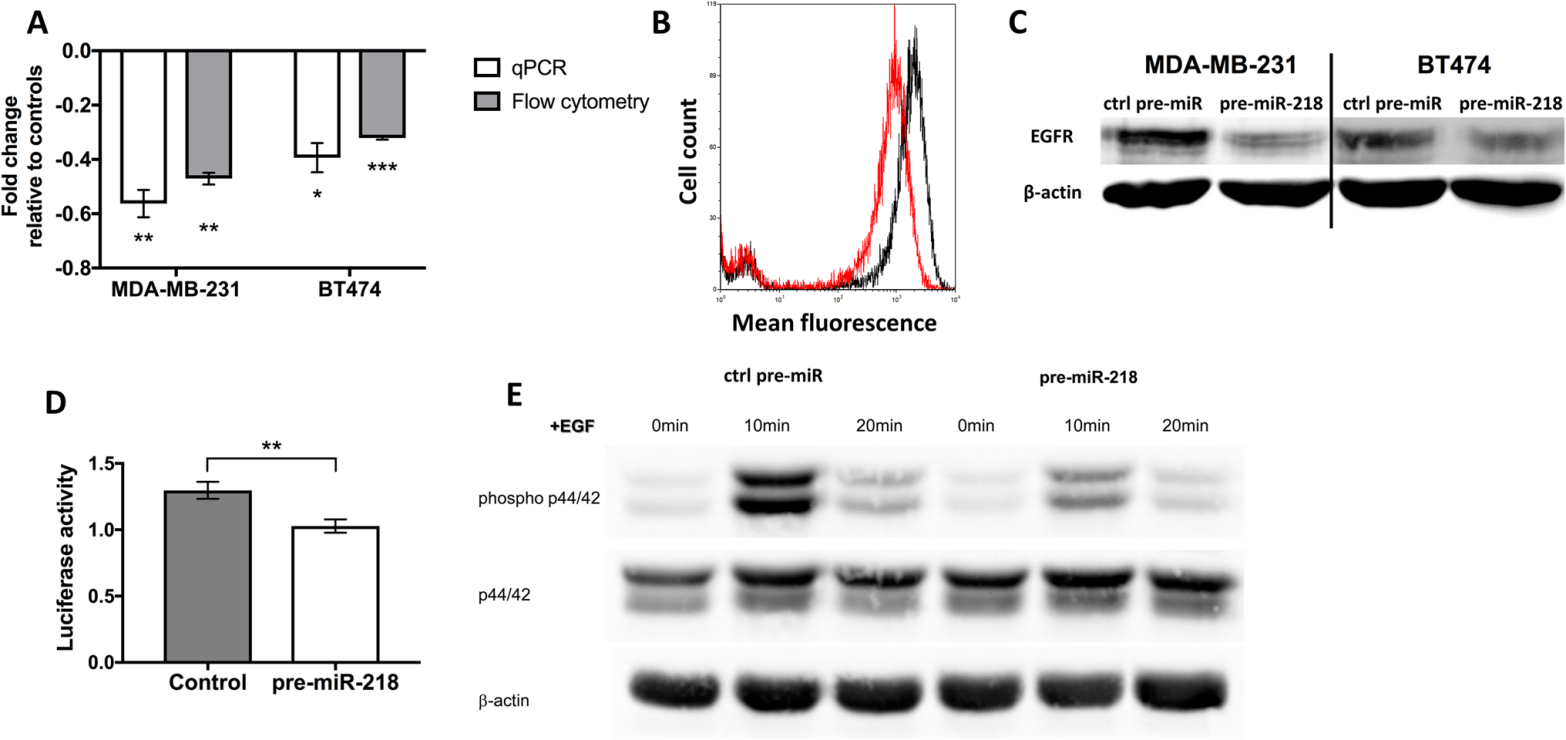
miR-218 overexpression targets EGFR expression and the p44/42 MAPK. **A** After miR-218 overexpression, EGFR levels were decreased in breast cancer cell lines MDA-MB-231 and BT474 in qPCR and flow cytometry analyses. **B** Representative flow cytometric analysis in MDA-MB-231 cells in controls (black) and pre-miR-218-transfected cells (red). **C** pre-miR-218 transfection also decreased EGFR levels in western blots. **D** Loss of luciferase activity in pre-miR-218-transfected cells relative to control cells. **E** Decrease in EGF-induced p44/42 phosphorylation after pre-miR-218 compared to control cells. *p < 0.05; **p < 0.01; ***p < 0.001

Following our analyses, we performed real-time qPCR testing after pre-miR-218 or negative control pre-miR treatment. In the breast cancer cell lines MDA-MB-231 and BT474, *EGFR* was significantly reduced 24 h after pre-miR-218 transfection (*p* = 0.002 and *p* = 0.018, respectively, Fig. 2A, white bars).

We then verified downregulation of EGFR at the protein level 72 h post transfection. EGFR protein levels were decreased in both cell lines after miR-218 overexpression using flow cytometric (Fig. 2A and B) and western blot (Fig. 2C) and analyses. Data indicated a substantial loss of EGFR levels in both cell lines (47% in MDA-MB-231, mean fluorescence activity 108.0 vs. 55.6; and 32% in BT474 cells, mean fluorescence activity 4.47 vs. 3.06; *p* = 0.002 and *p* < 0.001, respectively). A representative measurement from MDA-MB-231 cells is shown in Fig. 2B.

To verify whether EGFR is a direct target for miR-218, we performed a luciferase assay, where co-transfection of an *EGFR* 3′UTR binding site expressing reporter plasmid together with the miR-218 precursors was performed. Compared to the negative control pre-miR, the pre-miR-218-transfected cells showed a significant 25% decrease of normalized luciferase activity in MDA-MB-231 cells 72 h after transfection (Fig. 2D).

Given the now-established influence of miR-218 on EGFR expression, we sought to investigate subsequent downstream effects. The primary downstream effector of EGFR is the p44/42 MAPK, which becomes phosphorylated due to EGFR activation upon binding to its ligand EGF. While the level of total p44/42 protein was not affected by miR-218, the phosphorylation of p44/42 was decreased at 10 min and 20 min after stimulation with 100 ng EGF in the pre-miR-218-transfected MDA-MB-231 cells compared to the controls (Fig. 2E).

### miR-218 overexpression influences individual cell growth, movement, cell division & radiation resistance

**Fig. 3:**
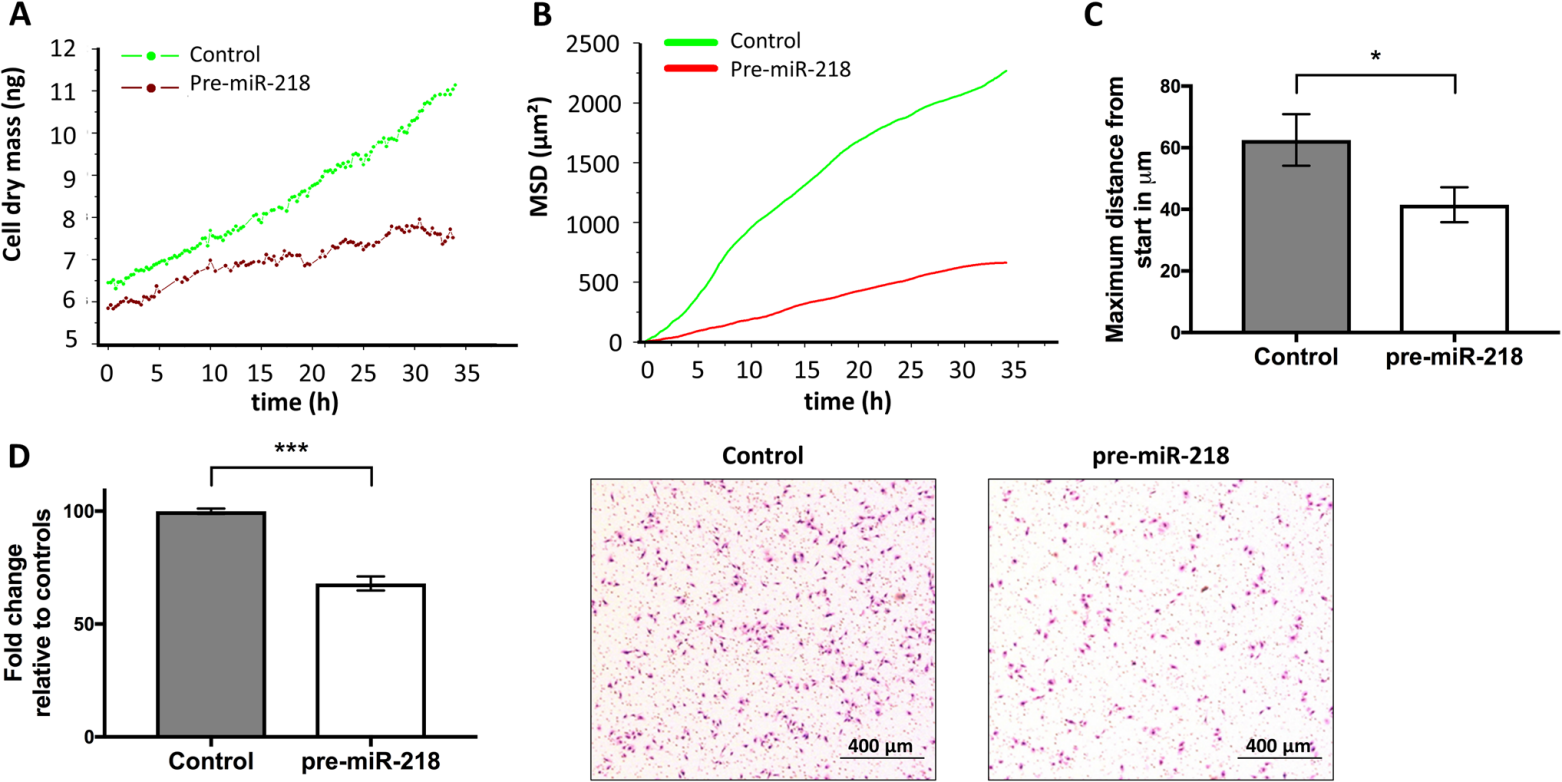
pre-miR-218 transfection substantially reduces individual cell traits in MDA-MB-231 cells. **A** Cell dry mass remains stagnant over time in pre-miR-218-transfected cells while increasing in control miR-transfected cells. **B** Mean squared displacement (MSD) of control and pre-miR-218 treated cells. **C** Maximum distance from start point in control and pre-miR-218-transfected cells. **D** Invasiveness is strongly reduced in pre-miR-218 treated cells relative to controls. *p < 0.05; ***p < 0.001

Multiple studies used colony formation or CCK8 assays to determine changes in proliferation (Li et al. 2012; Liu et al. 2016; Zhao et al. 2017; Han et al. 2019; Xia et al. 2019) and wound healing investigations to investigate changes in migration (Liu et al. 2016; Han et al. 2019), reporting anti-tumorigenic outcomes for miR-218 high expressing cells. Given the influence of EGFR on these outcomes, we aimed to substantiate these findings by performing single-cell analyses via time-lapse video microscopy. Here, we found that cell dry mass (Fig. 3A) of the pre-miR-218-transfected cells remained nearly stagnant over time whereas this parameter substantially increased in control pre-miR transfected cells. Additionally, single cell tracking revealed a decrease in motility of the miR-218-overexpressing cells compared to the controls, as determined by measuring the mean squared displacement (MSD, Fig. 3B) and the maximum distance of control and pre-miR-218-transfected cells from the starting point (*p* = 0.04, Fig. 3C). Finally, invasiveness in vitro was significantly reduced after pre-miR-218 transfection (*p* < 0.001, Fig. 3D, representative panels for control and pre-miR-218 cells are also shown).

**Fig. 4:**
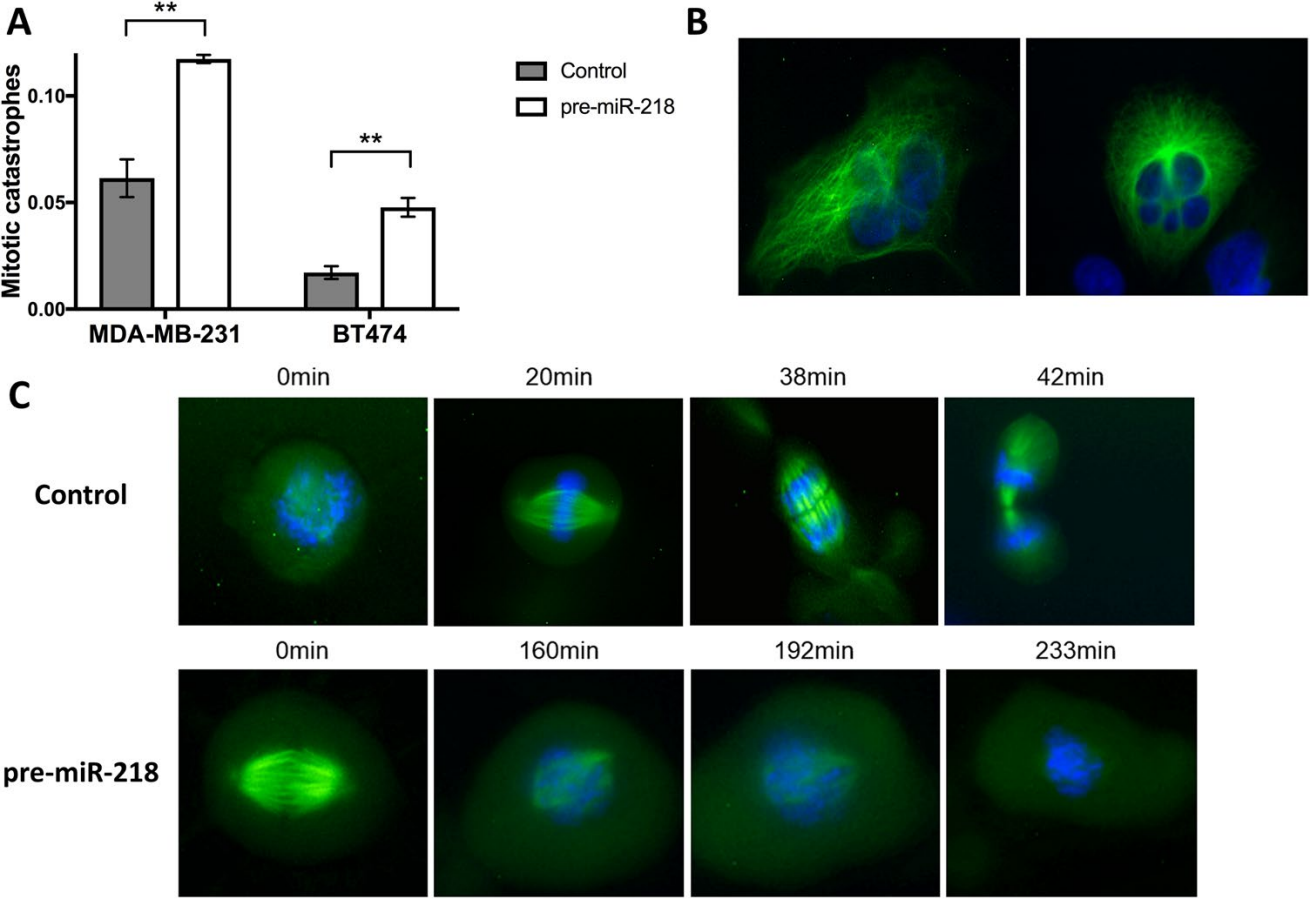
Cell division in pre-miR-218-transfected cells. **A** Mitotic catastrophes increase in MDA-MB-231 and BT474 cells after pre-miR-218 transfection. **B** Polynucleated cells are documented after pre-miR-218 transfection. **C** Cell division is prolonged and impaired in this example of a miR-218 overexpressing cell compared to a control cell. **p < 0.001﻿

Fluorescence video microscopy helped determine cell division, with Tubulin-GFP BacMam2.0 (Invitrogen) staining tubulin, and Hoechst 33342 acting as a nuclear dye. While the control-pre-miR treated cells showed a normal chromosome distribution and division time, numerous pre-miR-218 overexpressing MDA-MB-231 or BT474 cells were not able to divide correctly. This persisted over a longer time resulting in an increased proportion of cells showing mitotic catastrophes (Fig. 4A). Notably, miR-218 overexpression led to morphologically polynucleated cells (Fig. 4B). We also documented the successful cell division in control cells and the unsuccessful mitotic catastrophe in miR-218 overexpressing cells microscopically over time (Fig. 4C, video file provided as Supplementary Video).

**Fig. 5:**
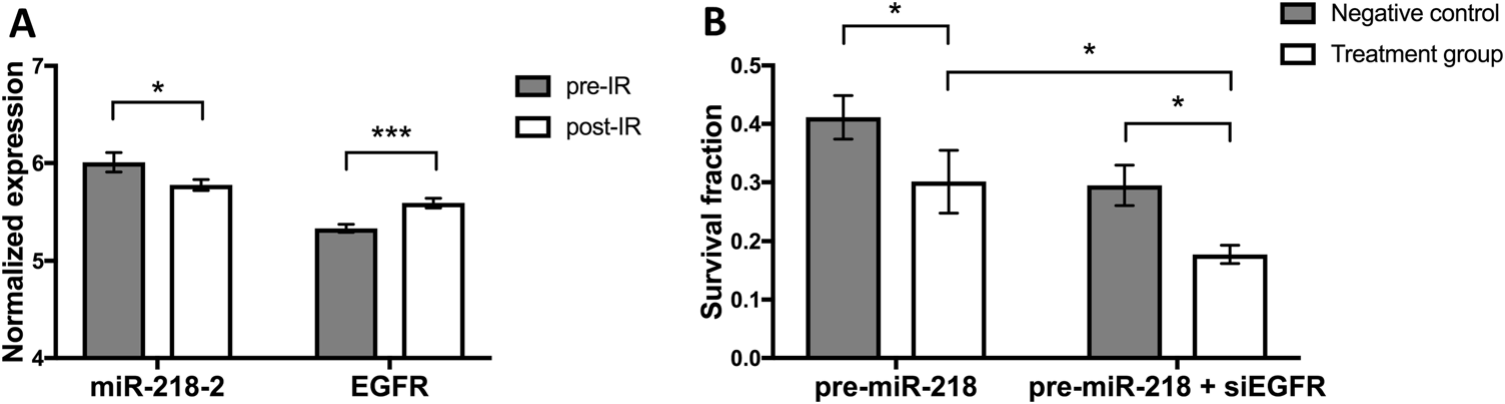
miR-218 and EGFR determine radiation response in breast cancer. **A** In paired analyses from pre- and post-irradiation breast cancer specimens from the GSE65505 dataset, EGFR levels were increased. Meanwhile, miR-218–2, the most common miR-218 isoform quantified in the analysis, was reduced. **B** In clonogenic analyses, pre-miR-218-transfected MDA-MB-468 cells showed reduced survival fractions compared to controls. Survival rates were even lower in cells that underwent pre-miR-218 transfection as well as silencing of EGFR. *p < 0.05; ***p < 0.001

Given that EGFR has been associated with radioresistance, we hypothesized that miR-218 may also influence radiation response. In a sample of pre- and post-radiotherapy specimens from the GSE65505 dataset, we found that EGFR was increased after irradiation while levels of the most common miR-218 isoform quantified in the analyses, miR-218–2, were decreased (Fig. 5A). We subsequently assessed clonogenic potential in MDA-MB-468 breast cancer cells after a radiation dose of 2 Gy. Pre-miR-218 overexpressing cells showed reduced survival after irradiation compared to controls. This effect was significantly increased in cells undergoing additional EGFR silencing (Fig. 5B).

## Discussion

**Fig. 6:**
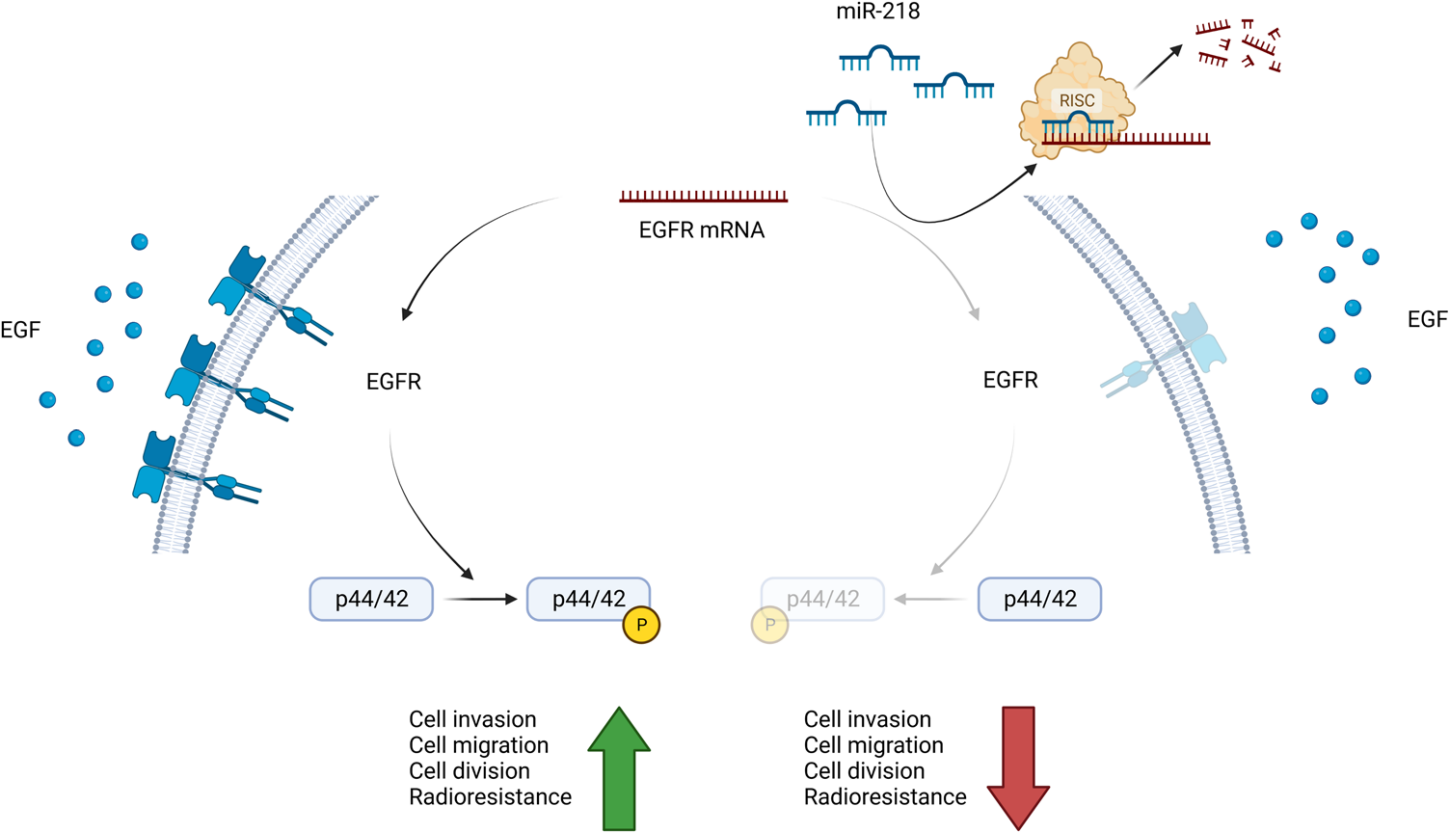
Summary of findings. *EGFR* epidermal growth factor receptor, *mRNA* messenger ribonucleic acid, *RISC* RNA-induced silencing complex. The figure was created with biorender.com

In the present study, we demonstrated that miR-218 directly targets *EGFR* mRNA in TNBC, resulting in antagonistic expression patterns in vitro and in vivo. We additionally found that miR-218 overexpression resulted in decreased cell proliferation, motility, and invasiveness, but increased mitotic catastrophes. Finally, radioresistance was decreased prominently not only after miR-218 overexpression and EGFR silencing combined, but also after miR-218 overexpression alone. Findings are summarized in Fig. 6.

### The miR-218-EGFR relationship

EGFR has previously been described to be a direct target of multiple micro RNAs, including miR-7 and miR-206. Additionally, close to a dozen micro RNAs target the EGFR pathway in general (Han et al. 2015). While bioinformatic analyses suggest that more than 100 micro RNAs may directly target *EGFR*, only few of these predicted interactions have been validated experimentally (Chan et al. 2012).

Our findings indicate a direct binding event between miR-218 and *EGFR*. They are in line with previous studies in different tumor entities. In lung cancer, two groups demonstrated direct binding between miR-218 and the 3′UTR region of the *EGFR* mRNA (Zhu et al. 2016; Islam et al. 2023).

Interestingly, our findings suggest that the miR-218-*EGFR* mRNA binding event goes beyond translational repression, as we found EGFR protein and mRNA levels repressed. This implies that mRNA decay is also initiated as part of the binding process.

Notably, we found a strong downregulation of the *EGFR* mRNA despite only moderate binding prediction from the TargetScan algorithm. This is not surprising as previous studies have shown that extensive complementarity is not a prerequisite for micro RNA target mRNA degradation (Mohr and Mott 2015). There is no specific evidence as to how the *EGFR* mRNA decay takes place. However, miR-218 is known to be part of a RNA-induced silencing complex (RISC) to facilitate mRNA cleavage (Thiebes et al. 2015). Hence, we hypothesize that miR-218 directly targets and cleaves the *EGFR* mRNA via RISC formation.

Antagonistic relationships between miR-218 and EGFR have also been demonstrated in glioblastoma (Mathew et al. 2015) and, indirectly, in osteosarcoma (Lin et al. 2020) and esophageal squamous cell carcinoma (Qu et al. 2020). Conversely, a recent preprint found a co-expression of miR-218 and EGFR in breast cancer, hypothesizing that EGFR is indirectly upregulated via miR-218 (Qian et al. 2021). However, this is the only investigation to suggest a positive correlation between these factors, while our breast cancer study—and the remaining literature from other tumor entities—disagree.

There is evidence that links high miR-218 expression to prolonged survival in breast cancer patients (Liu et al. 2015; Ahmadinejad et al. 2017; Setijono et al. 2018). A small investigation from 32 patients also found a differential expression between different grades (Ahmadinejad et al. 2017). Leveraging the availability of large-scale patient gene expression data, we found that miR-218 is overexpressed in low-grade, ER-positive tumors—a good-prognosis group. Conversely, EGFR was most prominently expressed in high-grade ER-negative samples in our study and is a known marker of poor prognosis in breast cancer (Richard et al. 1987). Interestingly, miR-218 was found to be a negative prognostic marker in cervical cancer (Cruz‐De la Rosa et al. 2022) despite its role as a positive prognostic marker in breast cancer. This underlines the need for individualized analyses between different tumor entities.

We also tried to identify effects downstream of EGFR expression after miR-218 transfection and found that phosphorylation of p44/42 was decreased after EGF stimulation in miR-218 overexpressing cells. This supports reduced EGFR activity as EGFR activation leads to phosphorylation of p44/42 (Sakai et al. 2006). It underscores the functional relevance of lower EGFR expression after miR-218 transfection.

### Pre-miR-218 transfection limits cell growth and motility—findings from a digital holographic microscopy investigation

Given the importance of EGFR signaling for cell cycle progress and proliferation, we decided to use digital holographic microscopy techniques to uncover subsequent changes. Our findings of reduced cell dry mass underline previous studies that reported reduced colony formation and cell viability in miR-218 overexpressing cells (Li et al. 2012; Liu et al. 2016; Zhao et al. 2017; Han et al. 2019; Xia et al. 2019). Our experiments additionally allow for long-time monitoring of individual cellular parameters given constant readouts over more than 30 h. We show a steady and consistent reduction in cell dry mass. This indicates that cells do not only decrease proliferation, but also lose the ability to grow in size and weight, indicating reduced cell metabolism. Meanwhile, cell division seems to be substantially impaired considering the increase in mitotic catastrophes seen after pre-miR-218 overexpression. A study in lung cancer points to a mechanistic connection between miR-218 and mitotic instability via TDP52 (Kumamoto et al. 2016). Previous studies have additionally indicated an increase in apoptosis after miR-218 overexpression (Zarogoulidis et al. 2015; Zhang et al. 2018). Our study identifies mitotic catastrophe as one key anti-proliferative pro-apoptotic mechanism.

We also see that individual cells move significantly less, providing more granular data compared to previous wound healing assay findings (Liu et al. 2016; Han et al. 2019). Decreased migration in individual cells is another key anti-tumorigenic characteristic.

Finally, cell invasion is reduced after pre-miR-218 overexpression. This supports ongoing debate between some studies reporting reduced metastatic ability in miR-218 overexpressing cells (Yang et al. 2012; Han et al. 2019), while others demonstrated increased bone metastasis formation in miR-218 high-expressing tumors (Hassan et al. 2012; Taipaleenmäki et al. 2016).

### Combined miR-218 overexpression and EGFR knockdown sensitize cells to irradiation

We found miR-218 decreased after irradiation, while EGFR levels increased. We believe that both expression changes indicate induced DNA damage repair or resistance to radiotherapy. We subsequently targeted both expression changes in vitro. In line with previous literature (Hu et al. 2019) we found that miR-218 overexpression sensitized cells to irradiation. EGFR itself is also known to be a marker of radioresistance (Gee and Nicholson 2003). Interestingly, cell survival after radiotherapy was additionally reduced if miR-218 overexpression was combined with artificial EGFR knockdown. The combination of both treatments seems to most effectively reduce radioresistance, underlining the synergistic function of EGFR downregulation and miR-218 overexpression.

There are some limitations to this study. First, not all experiments were performed in two different breast cancer cell lines. However, key mechanistic experiments relied on data from two cell lines. Second, we did not specifically investigate EGFR-mutant cancers and are thus unable to draw conclusions regarding this group. Third, while some data relies on primary patient data, the larger part of this study is limited to in vitro experimentation in TNBC cell lines, necessitating future in vivo confirmation. Fourth, we used the miRVana kit for all micro RNA qPCR analyses while the mRNA analyses were consistently performed with a different isolation kit. Future studies should consider using a single kit for miRNA and mRNA isolation, thus streamlining the workflow. Finally, the choice of housekeeping gene remains controversial. Our choices, *RNU6B* for micro RNA (Gee et al. 2011), especially in combination with use of the miRVana kit (Schindler et al. 2018), and *HPRT* for mRNA (De Kok et al. 2005) remain well-supported in the literature. However, there is no consensus regarding the optimal housekeeping gene for qPCR analyses and discussions are ongoing (Gorji-Bahri et al. 2021; Veryaskina et al. 2022).

## Conclusion

In the present study, we show that miR-218 and EGFR are inversely correlated in primary triple-negative breast cancer samples and cell lines. We subsequently demonstrate that miR-218 directly targets the Epidermal Growth Factor Receptor (EGFR) and results in increased mitotic catastrophes while cell dry mass and motility decrease. Finally, we demonstrate that miR-218 upregulation and EGFR knockdown both support radiosensitization, underlining the therapeutic relevance of the miR-218/EGFR pathway.

## Supplementary Information

Below is the link to the electronic supplementary material.

Supplementary file1 (DOCX 17 KB)

Supplementary file2 (PPTX 3319 KB)

## Data Availability

The authors confirm that the data supporting the findings of this study are available within the article and its supplementary materials.
