# Supplementary material for: Tumor suppressor miR-218 directly targets epidermal growth factor receptor (EGFR) expression in triple-negative breast cancer, sensitizing cells to irradiation
Source: J Cancer Res Clin Oncol. 2023 Apr 23;149(11):8455–65. doi: 10.1007/s00432-023-04750-x (PMC10374822; doi:10.1007/s00432-023-04750-x)
Supplement: Supplementary file 1 — Supplementary file1 (DOCX 17 KB) [file 432_2023_4750_MOESM1_ESM.docx]

**Supplementary Material**

**Supplementary Table S1.** siRNA or pre-miR used for experiments.

| **Target** | **Manufacturer** | **ID** |
| --- | --- | --- |
| *EGFR* | ThermoFisher Scientific | siRNA ID: s563 |
| Silencer™ Select Negative Control No. 1 siRNA | ThermoFisher Scientific | Catalog #: 4390844 |
| hsa-miR-218-5p | ThermoFisher Scientific | Assay ID: PM10328 |
| Pre-miR™ miRNA Precursor Molecules—Negative Control #2 | ThermoFisher Scientific | Catalog #: AM17111 |

**Supplementary Table S2.** Primers used for real-time PCR analysis.

| Gene | Primer sequence | Fragment length | Annealing temperature |
| --- | --- | --- | --- |
| *EGFR* | FW: GAGATCGCCACTGATGGAGG  REV: AACCCCGAGGGCAAATACAG | 272 bp | 60 °C |
| Gene | **Manufacturer** | **Catalog #** | |
| *HPRT* | Qiagen | QT00059066 | |
| hsa-miR-218 | ThermoFisher Scientific | Assay ID: 000521 | |
| *RNU6B* | ThermoFisher Scientific | Assay ID: 001093 | |

**Supplementary Table S3**. Sequence, position and PCT value of the predicted seed site for miR-218 regarding the *EGFR* mRNA.

| **GENE** | **SEQUENCE** | | | | |  |  |  |  |  |  |  |  |  |  |  |  |  |  |  |  |  |  |  |  |  |  | **P_CT_** |
| --- | --- | --- | --- | --- | --- | --- | --- | --- | --- | --- | --- | --- | --- | --- | --- | --- | --- | --- | --- | --- | --- | --- | --- | --- | --- | --- | --- | --- |
| ***EGFR*** 3´ UTR |  |  |  |  |  |  |  |  |  |  |  |  |  |  |  |  |  |  |  |  |  |  |  |  |  |  |  |  |
| 195-201 | 5´ | A | C | C | A | G | A | U | G | A | U | U | G | U | U | C | A | A | A | G | C | A | C | A | G | A | 3´ |  |
|  |  |  |  |  |  |  |  |  |  |  |  |  |  |  |  |  |  | I | I | I | I | I | I | I |  |  |  | <0,1 |
| miR-218 | 3´ |  |  |  | U | G | U | A | C | C | A | A | U | C | U | A | G | U | U | C | G | U | G | U | U |  | 5´ |  |
