# Supplementary figures and images for: Tumor suppressor miR-218 directly targets epidermal growth factor receptor (EGFR) expression in triple-negative breast cancer, sensitizing cells to irradiation
Source: J Cancer Res Clin Oncol. 2023 Apr 23;149(11):8455–65. doi: 10.1007/s00432-023-04750-x (PMC10374822; doi:10.1007/s00432-023-04750-x)

## Slide 1
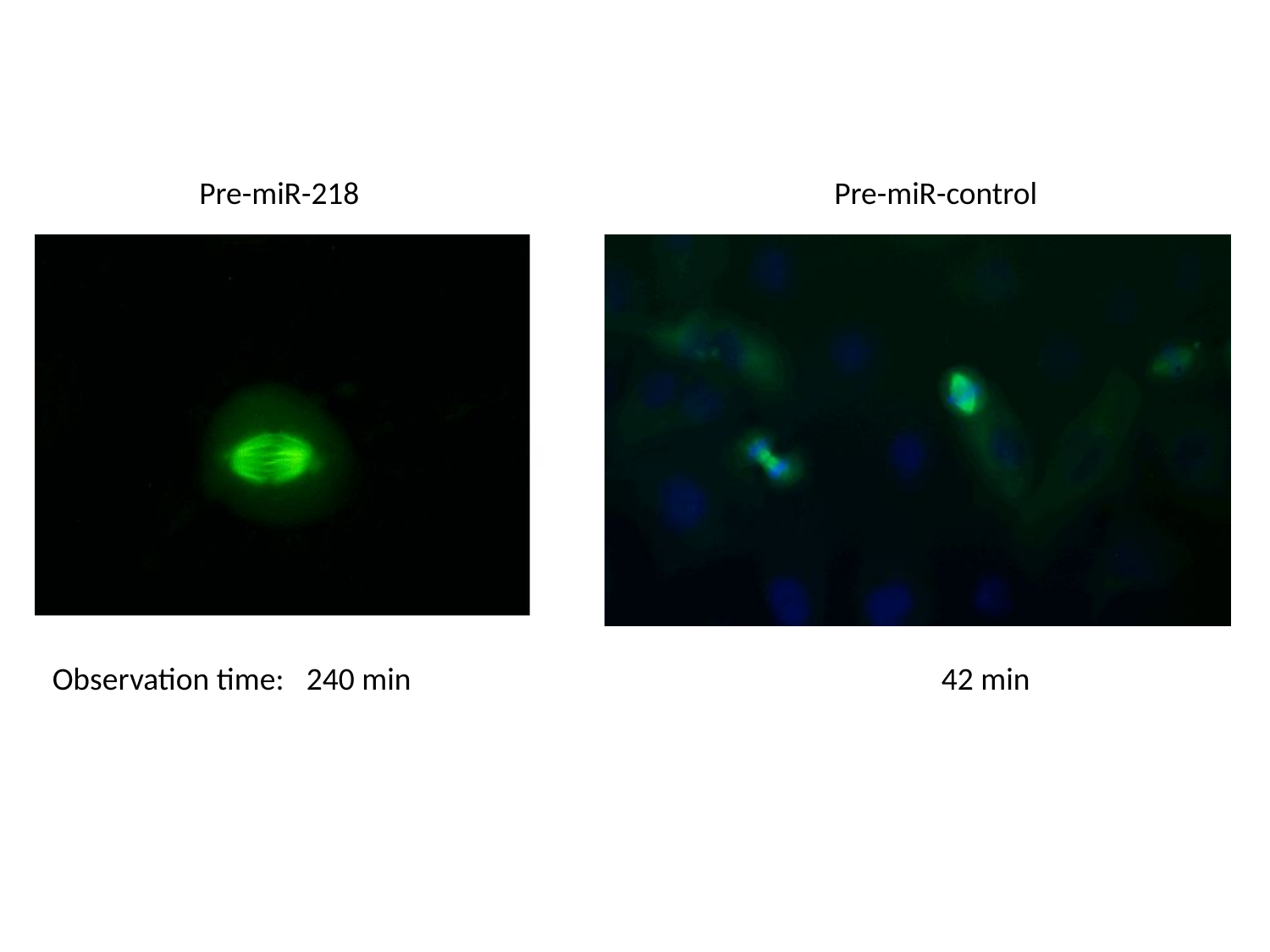

Pre-miR-218				Pre-miR-control
Observation time: 	240 min					42 min

Supplement: Supplementary file 2 — Supplementary file2 (PPTX 3319 KB) [file 432_2023_4750_MOESM2_ESM.pptx]
